# Prevalence and Significance of Incidental PET/CT Findings of Cancer Detected in Patients Evaluated for Their Primary Hematologic Malignancy: A Systematic Review

**DOI:** 10.3390/curroncol31120550

**Published:** 2024-11-24

**Authors:** Jessie Luo, Nizar J. Bahlis, Denise Chan, Peter Duggan, Victor H. Jimenez-Zepeda, Holly Lee, Sylvia McCulloch, Paola Neri, Jason Tay

**Affiliations:** 1 Department of Microbiology and Immunology, University of British Columbia, Vancouver, BC V6T 1Z4, Canada; 2 Department of Medical Oncology and Hematology, Tom Baker Cancer Center, Calgary, AB T2N 4N2, Canadasylvia.mcculloch@albertahealthservices.ca (S.M.);; 3 Arnie Charbonneau Cancer Institute, Calgary, AB T2N 4Z6, Canada; 4 Department of Radiology, University of Calgary, Calgary, AB T2N 4Z5, Canada

**Keywords:** PET/CT, hematologic malignancies, incidental findings, myeloma/plasma cell disorder, lymphoproliferative diseases

## Abstract

In the evaluation of a patient’s primary hematologic malignancy, positron emission tomography/computed tomography (PET/CT) imaging may incidentally detect a concerning abnormality suggestive of a second concurrent cancer. Despite accounting for nearly 10% of all cancers diagnosed in Canada, there has yet to be a systematic review focused on the prevalence and significance of these incidental PET/CT findings in the context of primary hematologic malignancies. As such, a systematic search strategy was employed on MEDLINE and Embase to document the prevalence and clinical significance of incidental PET/CT findings suggestive of a second concurrent cancer detected in patients evaluated for their primary hematologic malignancy. Thirteen studies published between 2008 and 2022 were reviewed, including conference abstracts (*n* = 8) and journal articles (*n* = 5). Clinically significant incidental cancers were detected with a median of 2.4% (range: 1.1–10.3%) in patients with myeloma/plasma cell disorders, compared to a median of 1.5% (range: 0.3–2.8%) in patients with lymphoproliferative diseases. The most common anatomic regions of clinically significant incidental malignancies were identified in the gastrointestinal tract (44.4%), followed by the thyroid gland (22.2%) and lungs (7.9%). In most cases, early detection of incidental cancers led to successful early interventions. PET/CT scans occasionally identify second primary malignancies that require additional attention. These findings may affect the treatment of a patient’s primary hematologic malignancy, and as such, timely coordinated management is important for improved outcomes. This review may inform physicians and administrators of the risk of incidental second malignancies and may highlight a need for enhanced cancer treatment pathways.

## 1. Introduction

Hematologic malignancies include lymphomas (Hodgkin’s, non-Hodgkin’s), leukemias (acute lymphocytic, chronic lymphocytic, acute myeloid), myelomas, myelodysplastic syndromes, and myeloproliferative neoplasms [1], accounting for nearly 10% of all cancers diagnosed in Canada [2]. In economically developed nations, they represent the fourth most common group of cancer diagnoses in men and women [3].

Positron emission tomography/computed tomography (PET/CT) is an imaging modality that is used to evaluate the extent of cancer [4]. It utilizes PET imaging to detect functional abnormalities in the body in conjunction with CT scans to provide an anatomic visualization of the structures involved in radioactive uptake [5]. In lymphoma care, identification of limited or more extensive disease by PET/CT scans at diagnosis or mid-treatment helps clinicians choose the appropriate therapy, as well as the duration of therapy. In contrast, in myeloma care, PET/CT can help make a diagnosis of symptomatic myeloma in lieu of a whole-body MRI scan as per International Myeloma Working Group (IMWG) criteria for diagnosis of symptomatic myeloma. Additionally, it aids in identifying potentially isolated sites of disease for plasmacytoma that may be amenable to radiation therapy. However, beyond making a diagnosis of symptomatic myeloma, the prescribed type and duration of systemic treatment are unlikely to be altered.

When PET/CT scans are used in the staging and treatment planning of a patient’s primary cancer, they may detect abnormalities suggestive of a second concurrent cancer unrelated to the primary diagnosis. A biopsy for pathological diagnosis is often required to confirm the clinical significance of these incidental findings. The assessment of incidental cancer findings impacts the appropriateness and timing of therapies for the primary diagnosis. As such, insight into the prevalence and clinical significance of incidental PET/CT findings for concurrent cancers is essential for optimizing patient management.

Previous studies estimate that the prevalence of clinically significant incidental second malignancies is 1.2–1.7% in general oncology [6,7,8]. However, it is not clear how hematologic malignancy patients compare to this as there has yet to be a comprehensive review to synthesize this information. This knowledge gap may highlight the uncertainty of (1) Whether incidental cancer findings are a frequent concern in the context of hematologic malignancies. (2) Whether incidental findings are commonly benign or malignant. (3) Whether there is a need for improved clinical pathways to address incidental findings in patients with a primary hematologic malignancy.

The current systematic review aims to summarize the current literature and fill the knowledge gaps mentioned above. Specifically, the findings may provide an estimate for the prevalence of incidental second cancers in patients with hematologic malignancies and the context of how they were identified. By assessing the current literature, we may identify risk factors or trends in patients with concurrent cancers and highlight areas for further research.

## 2. Materials and Methods

### 2.1. Protocol Registration

The systematic review protocol was registered in PROSPERO (CRD42023441646) on 17 July 2023. It was designed in compliance with Preferred Reporting Items for Systematic Review and Meta-Analysis Protocols (PRISMA-P) guidelines.

### 2.2. Systematic Review Design

A systematic review was performed by searching for studies relating to the prevalence and clinical significance of incidental PET/CT findings in patients with hematologic malignancies. This study was conducted following Preferred Reporting Items for Systematic Reviews and Meta-analyses (PRISMA) guidelines.

#### 2.2.1. Source Eligibility

Sources were assessed for eligibility according to Table 1.

#### 2.2.2. Search Strategy

A literature search strategy (refer to Appendix A) was employed on MEDLINE and Embase through the Ovid interface. Sources were searched from the inception of the databases to 26 July 2024, in which database coverage started from 1946 on MEDLINE and 1974 on Embase. The reference lists of all eligible studies and relevant systematic reviews identified from the databases were searched for additional sources. The search strategy was designed with the help of a librarian experienced with literature searches. Search fields were based on medical subject heading (MeSH) terms and relevant keywords. Searches were limited to the English language due to limitations in accurate translation resources available. However, the location of the publication was not restricted.

#### 2.2.3. Study Selection

Before the screening process, duplicates and retracted articles were removed. The screening of references obtained from the databases occurred in two stages. In stage one, the titles and abstracts of all references were screened for relevancy. This stage was conducted by one reviewer (JL) due to time and resource limitations. References without abstracts were selected based on the full texts unless exclusion could be determined solely from the title. To ensure that relevant studies were not excluded due to uncertainty in the content of the abstracts, these sources progressed to stage two of screening. In stage two, the full texts of all potentially

Eligible studies were screened by two independent reviewers (JL and JT) until a consensus was reached. The selection process was summarized according to PRISMA flowchart guidelines [9].

#### 2.2.4. Data Collection

One reviewer (JL) extracted relevant data, and another reviewer (JT) verified the collected data items. Any discrepancies in data extraction were discussed until a consensus was reached. Data were extracted for the following domains where available:

- **Study characteristics**: author names, date of publication, journal of publication, study location country or institution, and source of data.
- **Population characteristics**: primary cancer type, sample size, age—mean age and/or age range, number of male and female participants, and time period of PET/CT scans.
- **Methods**: type of study, imaging techniques used, and if PET/CT scans were collected consecutively.
- **Primary outcomes**: prevalence of incidental findings, clinical significance, and incidental cancer type.
- **Secondary outcomes**: anatomic regions associated with benign or malignant incidental findings, correlations between SUV max value and identification of second malignancy, correlations between size of PET/CT abnormality and identification of second malignancy, correlation between stage of primary malignancy and prevalence of incidental findings

#### 2.2.5. Risk of Bias

No specific risk-of-bias evaluation tools were identified for the needs of this systematic review. Thus, a modified tool was created that incorporates elements of two relevant tools: ROBINS-I (Risk Of Bias In Non-Randomized Studies-of Interventions) and QUADAS-2 (Quality Assessment of Diagnostic Accuracy Studies). This modified tool evaluated the three domains of patient selection, missing data, and selection of reported results. Domains such as confounding and measurement of outcomes were considered but deemed not applicable to the current systematic review. The results of our risk-of-bias assessment were visualized using the Risk-of-bias VISualization (robvis) web app [10].

#### 2.2.6. Data Synthesis

Data and statistics were reported quantitatively. Due to data heterogeneity, no meta-analysis was conducted. Data were grouped based on the primary hematologic malignancy.

## 3. Results

**Figure 1:**
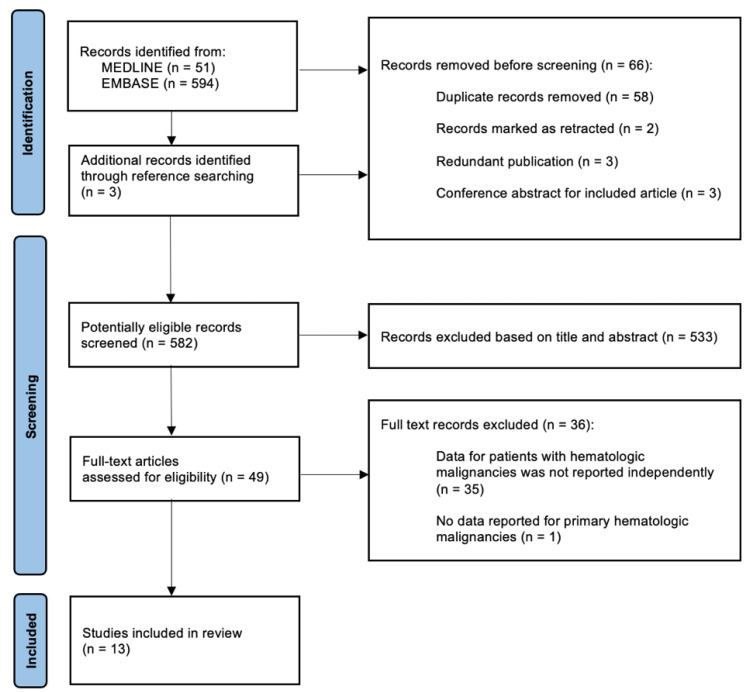
Systematic review flowchart of the screening and selection processes. Flowchart format adapted from PRISMA guidelines [9].

The screening and selection process is depicted in Figure 1. We identified 648 studies, in which 582 potentially relevant studies were screened after the removal of duplicates, retracted articles, redundant publications, and redundant conference abstracts. After screening for the titles and abstracts, 533 studies were removed and 49 proceeded to the full-text evaluation. Following the full-text screening, an additional 36 studies were excluded. Common reasons for exclusion included the absence of subgroup data for patients with hematologic malignancies not being reported independently or a lack of data reported for primary hematologic malignancies. Finally, 13 studies were included for analysis [11,12,13,14,15,16,17,18,19,20,21,22,23]. 

### 3.1. Study Characteristics

A summary of study characteristics is included in Table 2. All studies were published between 2008 and 2022, with 46% (*n* = 6) published in 2018–2022, 23% (*n* = 3) in 2013–2017, and 31% (*n* = 4) in 2008–2012. A majority of studies were conducted in Europe (54%, *n* = 7) and North America (31%, *n* = 4), specifically the United States.

The median sample size of included studies was 130, ranging from 29 to 1868. Most studies were conference abstracts (62%, *n* = 8) and the remaining were journal articles (38%, *n* = 5). While FDG PET/CT was the main imaging modality used in studies (77%, *n* = 10), one study examined scans from either PET/CT or CT imaging [20], and two studies did not specify the PET radiotracers used [14,23]. The primary hematologic malignancy reviewed was myeloma and plasma cell disorders in 58% (*n* = 7) of sources, while 33% (*n* = 4) studied lymphoproliferative disorders. One study included both myeloma and lymphoma patients. Most studies looked at the incidental findings of all anatomical regions (*n* = 10) while two studies specifically focused on incidental findings of the gastrointestinal tract or thyroid.

### 3.2. Primary Outcome—Prevalence of Secondary Malignancies

A summary of the prevalence of suspicious or clinically significant incidental malignancies is synthesized in Table 3. A total of 63 clinically significant incidental malignancies were detected amongst the population of the identified studies (*n* = 4450), 39 of which were biopsy-proven or pathologically determined. The most frequent anatomic regions of clinically significant incidental malignancies were the gastrointestinal tract (44.4%), the thyroid (22.2%), the lungs (7.9%), and the breast (4.8%). Clinically significant malignancies were also found in the pancreas and prostate, at 3.2% each. Among patients with myeloma/plasma cell disorders, a median of 2.4% (range: 1.1–10.3%) were diagnosed with a significant incidental malignancy, determined either clinically or by biopsy. Similarly, a median of 1.5% (range: 0.3–2.8%) of patients with lymphoproliferative disorders were diagnosed with a significant incidental malignancy either clinically or by biopsy.

We examined the potential impact of study characteristics on our results. Based on overlapping ranges, none of the study characteristics made a significant difference in the prevalence of malignant incidental findings. For instance, there was no obvious difference between patients examined in Europe (Median: 3%; range 1.2–10.3%) and North America (Median: 1.3%, range: 1.1–1.6%). Studies with smaller sample sizes (median: 1.9%, range: 1.1–10.3%), defined as less than the median of 130 patients, did not have a significantly higher prevalence of clinically significant incidental malignancies than larger studies (median: 1.6%, range: 0.3–4.2%) with a sample size of at least 130. However, there appears to be a slight increase in prevalence over the periods of 2008–2012 (median: 1.5%, range: 0.3–2.8%), 2013–2017 (median: 3.0%, range: 1.1–4.2%), and 2018–2022 (median: 2.0%, range: 1.1–10.3%), which may be attributed to the increase in accessibility or sensitivity of imaging technologies.

### 3.3. Secondary Outcomes

Four studies discussed the correlation between SUVmax and disease characterization [12,17,18,23]. SUVmax is a measure of radiological uptake in PET scans, which can be indicative of abnormal activity. While a higher uptake could be expected to correlate to a higher risk of malignancy, three of the above studies concluded that differences in SUVmax were not able to distinguish between normal and abnormal findings [12,17,18]. On the other hand, one study suggests that an SUVmax of nine can be a threshold to differentiate PCNS lymphoma from incidental findings [23]. However, this value could not discriminate between incidental findings of non-malignant and malignant nature. Notably, another study found a positive correlation between SUVmax and the painfulness of lesions [12]. Among all included sources, there was little discussion on the correlation between malignancy size and clinical significance. However, one study found no significant difference between the mean sizes of benign and malignant nodules [18]. Taken together, these findings do not demonstrate a clear correlation between the size or activity of radiological abnormalities and disease severity or outcomes.

Interestingly, Sato et al. (2010) reported one case in which SUVmax may have been the only indicator of malignancy. Even with full remission of DLBCL, one patient was reported to have passed away from an incidental primary squamous cell carcinoma of the lung [19]. Serological tests for all lung tumour markers were within normal ranges and pulmonary symptoms were not observed during the mid-treatment PET/CT assessment for lymphoma. However, the retrospective analysis of the mid-treatment PET scans demonstrated an increase in SUVmax compared to the pre-treatment values, rising from 5.50 to 11.09. This retrospective observation may not be applicable in most cases as it was only reported in one out of the eight patients reviewed.

Due to the limitations of retrospective analyses, few studies discussed the follow-up of patient outcomes. However, one study found that patients without incidental findings experienced a higher overall survival than those with malignancy findings, though this was not statistically significant [20]. Additionally, three studies discussed the treatment of incidental malignancies [16,19,23]. Generally, treatments for suspicious findings were deemed successful. Among our reviewed studies, surgical procedures to remove incidental colon malignancies were particularly successful in preventing the progression and relapse of disease [19,23]. However, further follow-up of patient outcomes was not reported. Likewise, another study reported that early treatment of all patients with incidental second malignancies was successful [16]. However, details on the procedures performed and outcomes upon follow-up were not disclosed. Altogether, these observations suggest that early detection and timely management of incidental findings may lead to improved patient outcomes. However, more data are required to assess the impact of a variety of treatment plans on survival and clinical outcomes.

### 3.4. Risk of Bias Assessment

**Figure 2:**
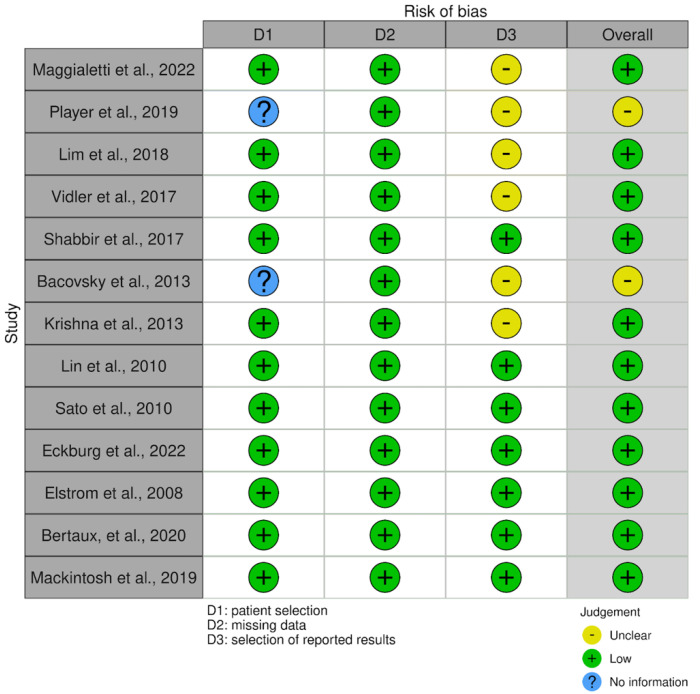
Risk of bias assessment chart for all included studies [11,12,13,14,15,16,17,18,19,20,21,22,23].

The risk of bias assessment is visualized in Figure 2. Low patient selection bias was observed as most studies collected data by selecting all patients within a specific timeframe and/or reviewing patient scans in a consecutive manner. Two studies provided little information on how patients were selected other than the patient characteristics. Bias due to missing data was not a prevalent concern as most studies were able to report and stratify their data into subgroups. Reporting bias was unclearly detected in six studies. This was due to the omission of some details about the determination of clinical significance. One study was unclear in reporting its data and inconsistently stated the number of incidentally detected primary cancers as 5% of patients and also 9 out of 240 patients [16]. Overall, the risk of bias was perceived to be acceptable across all included studies.

## 4. Discussion

Understanding the prevalence and clinical significance of incidental PET/CT findings suspicious for cancer is valuable information during the treatment of patients with a primary malignancy. These incidental findings often affect the timing of therapies for the primary cancer. As such, insight into the risk of malignancy and outcomes for patients with concurrent cancers may help clinicians manage these findings or improve the timeliness of clinical decision-making. While there is a larger range of literature on the prevalence of incidental findings, these reviews may be outdated and report total incidental findings without stratifying for patients with incidental second malignancies [24,25]. Moreover, there is a lack of literature focused on patients with primary hematologic malignancies. Therefore, this review contributes to these gaps in knowledge by providing insight into the current state of incidental findings in hem-oncology.

Our systematic review suggests that malignant incidental findings may be more prevalent in patients with myeloma/plasma cell disorders than those with lymphoproliferative diseases, with a median of 2.4% (range: 1.1–10.3%) and 1.5% (range: 0.3–2.8%), respectively. Notably, there is a large range of reported rates of PET/CT incidental findings. This may be related to individual and centre variations in practice, and perhaps reporting experience. However, our subgroup analysis did not demonstrate any differences between time cohorts, or European and U.S. studies. Additionally, our review did not reveal an association between SUVmax or size of abnormality with secondary malignancy.

A review of patient outcomes reveals that early detection of incidental cancers may lead to successful interventions when managing concurrent cancers. There were no cases where follow-up or treatment for a suspicious incidental finding negatively impacted the treatment of the patient’s primary cancer. On the contrary, one patient with complete remission of their primary hematologic malignancy was reported to have passed away due to their incidentally detected tumour [19].

Our review found that in lymphoma patients, incidental focal FDG uptake in the thyroid was associated with a 30% risk of malignancy while diffuse FDG uptake was associated with benign diagnoses [18]. This aligns with other studies where the risk of malignancy for incidental thyroid FDG uptake in patients with non-thyroid primary malignancies ranged from 28 to 42% [26,27]. Furthermore, our findings are in keeping with the general population, reporting a 27–36% risk of malignancy for focal FDG thyroid uptake and a 4–6% risk of malignancy for diffuse uptake [28,29,30,31]. While abnormal focal thyroid uptake detected by FDG PET is associated with a high risk of malignancy, patients with hematologic malignancies do not have an elevated relative risk of incidental thyroid malignancies as compared to general oncology or the general population.

Considering SUVmax with FDG PET can help identify areas of increased metabolic activity or inflammation. While some clinicians deem a threshold SUVmax of 2.5 as suspicious for a malignancy, others may disagree with this cut-off [32,33]. Indeed, the reality of differentiating malignant and benign findings is unsurprisingly nuanced as various factors impact the measurement and interpretation of SUV. For instance, some tumours are known to be FDG PET avid, such as squamous cell tumours [34]. Meanwhile, other tumours are not, such as renal cell cancer or low-grade tumours [35]. Our findings may highlight how optimizing the identification specificity and sensitivity of secondary cancers will ultimately require a combination of clinical judgement, radiology, and perhaps laboratory investigations, embraced by developed clinical prediction rules. Although such discussion is beyond the scope of our current review.

There can be nuances and variations in the practice of PET/CT interpretation. In lymphoma care, PET scans are often performed to identify other potential sites of disease, which may in turn influence therapy. In contrast, therapy remains similar in plasma cell disorders, with fewer changes based on PET results where the chosen chemotherapy more likely remains the same. For instance, patients with CNS lymphoma require additional scans such as PET/CT scans to rule out systemic disease where treatment is different. Perhaps this focus on disease sites that affect treatment decisions may lead to an underreporting of incidental malignancies in lymphoma studies, contributing to our findings that the prevalence of incidental PET/CT findings appears to be lower in lymphoma compared to plasma cell disorders.

There are limitations to our review that deserve mention. Firstly, our review may have missed articles or publications, and excluded non-English manuscripts. However, our systemic search criteria aimed to limit this bias. Second, the majority of the identified manuscripts were conference abstracts, limiting the available information. Third, due to the relatively limited number of studies and differences in reporting, robust summary statistics could not be provided. Instead, we were able to report on medians and ranges, and provide a narrative review. Indeed, most studies did not stratify the incidental findings suspicious for malignancy from the general findings, instead opting to report the prevalence of all incidental findings. In turn, we were unable to assess the proportion of suspicious findings that resulted in a clinically determined or biopsy-proven malignancy. However, in one study for patients with either myeloma or lymphoma, 32% (9/28 patients) of incidental findings suspicious for malignancy were pathologically determined to be malignant [20]. This suggests that incidental findings presenting concerns for a second concurrent cancer may be relevant to investigate as they could have a high risk of clinical significance. To corroborate this, one study affirmed the importance of investigating strongly suspicious incidental malignancies due to the high positive predictive value of 58% in findings requiring further work-up [15].

It was challenging to determine whether reported incidental lesions were of clinical or pathologic significance for secondary cancers. In the determination of clinical significance, 31% of studies (*n* = 4) declared that a biopsy was performed [15,17,18,19] while one study provided a statement of pathological findings [23], implying a pathological determination of clinical significance. In the other 62% of studies (*n* = 8), a biopsy was not explicitly declared. In one study, results were separated into benign and malignant categories, denoting that the determination of clinical significance was executed [22]. Another study retrieved data for whether a biopsy was performed, but that information was not reported for incidental findings [21]. In two studies [11,20], the determination of clinical significance was based on a significant impact on patient management and morbidity or mortality rather than pathology. In three studies [12,13,14], incidental malignancies were stated to be significant with no declaration of biopsy. This determination was based on the implication that the findings impacted patient management and further clinical decision-making. One study [14] stated that the findings were of “further pathology” without declaring pathological determination. Another study [16] described the diagnosed second malignancies without a declaration of biopsy or pathological determination. However, patients were surgically treated for their incidental findings, implying that clinically significant findings were deemed to impact patient management. The lack of detail on the process of determining the clinical significance of malignancies could be due to the limitations of conference abstracts. Generally, full-text articles were more likely to contain details on the decisions made by clinicians during the determination of clinical significance. For future studies, it would be helpful to have a standard reporting method to differentiate clinically suspicious incidental findings from biopsy-proven or clinically determined incidental malignancies.

Future studies should further examine the interplay between a patient’s primary hematologic malignancy and the incidental second primary malignancy. This could include reporting on delays in therapy associated with the investigation and treatment of the incidental finding, along with any subsequent patient outcomes. Such patient-centred outcomes can inform improvements in timely access to care when managing multiple patients with concurrent cancers.

## 5. Conclusions

Our review provides a summary of the current estimates of secondary cancers when PET/CT scans are performed in patients with hematologic malignancies. We suggest that this information can inform physicians and administrators of the risk of incidental second malignancies and highlight the need for enhanced cancer treatment pathways as the detection of incidental findings allows for early investigation of potentially significant concurrent diagnoses.

## Figures and Tables

**Table 1:** Eligibility criteria of sources.

Category	Description of Criteria
Population	Eligible studies should report data for patients with hematologic malignancies, including leukemias, lymphomas, myelomas, myelodysplastic syndromes, or myeloproliferative disorders. Study populations will not be restricted by age or cancer treatment stage. Studies that analyze data from all cancer patients may be included if the outcomes for patients with hematologic malignancies are reported independently.
Intervention	Eligible studies should analyze PET/CT scans. All PET/CT imaging modalities will be considered, such as 18-fluorodeoxyglucose (18F-FDG) PET/CT, combined PET and low-dose CT, or whole-body PET/CT.
Outcomes	Eligible studies should report the prevalence and/or clinical significance of incidental malignant findings. Although extracted data for primary outcomes will relate to incidental findings of a second concurrent cancer, eligible studies may report the prevalence of oncologic or non-oncologic incidental findings as it is possible that some studies did not detect any incidental malignancies at all.
Study design	Retrospective studies, case series, and conference abstracts will be included. Case reports, previous systematic reviews, and non-English studies will be excluded.

**Table 2:** Summary of study characteristics. High-grade lymphomas include Sezary syndrome, diffuse large B-cell lymphoma (DLBCL), mantle cell lymphoma, anaplastic lymphoma, and CNS lymphoma. Low-grade lymphomas include follicular and marginal zone lymphomas.

Study	Primary Diagnosis	Country	Type of Publication	Sample Size	Purpose of PET/CT
Maggialetti et al., 2022 [11]	Multiple myeloma	Italy	Article	112 patients	Staging (*n* = 28) or treatment response assessment (*n* = 84)
Player et al., 2019 [12]	Multiple myeloma	U.K.	Conference abstract	29 patients	Diagnosis
Lim et al., 2018 [13]	Myeloma or plasmacytoma - IgG: 36 (42.9%) - IgA: 8 (9.5%) - no paraproteins: 12 (14.3%) - light chain band only on immunofixation: 28 (33.3%)	U.K.	Conference abstract	84 patients	Identification of bone disease in newly diagnosed or pre-existing myeloma or MGUS (*n* = 37) Identification of bone disease in newly diagnosed or pre-existing plasmacytoma (*n* = 17) Interim or post-treatment disease assessment (*n* = 30)
Vidler et al., 2017 [14]	Myeloma (21 IgG, 3 IgA, 2 non-secretory, 4 light chain disease, 2 biclonal) Solitary plasmacytoma = 1	U.K.	Conference abstract	33 patients	Diagnosis
Shabbir et al., 2017 [15]	Plasma cell dyscrasias	U.S.	Conference abstract	470 patients	Staging
Bacovsky et al., 2013 [16]	Multiple myeloma (*n* = 240) MGUS (*n* = 92)	Czech	Conference abstract	322 patients	Staging
Krishna et al., 2010 [17]	Multiple myeloma	U.S.	Conference abstract	729 scans	Staging
Lin et al., 2011 [18]	Lymphoma - NHL (*n* = 47) - HL (*n* = 5)	Australia	Article	1868 patients	For patients with incidental findings: Staging (*n* = 28), or therapy response assessment or restaging (*n* = 24)
Sato et al., 2010 [19]	Lymphoma - HL: 26 (9.0%), - NHL: 264 (91.0%) - High grade: 137 - Low grade: 79 - Other B-cell NHL (*n* = 28) - T or NK-cell NHL (*n* = 20)	Japan	Article	290 patients	Initial staging, restaging, mid-treatment response assessment, or post-therapy monitoring.
Eckburg et al., 2022 [20]	Cutaneous T cell lymphoma; Sezary syndrome, mycosis fungoides	U.S.	Article	88 patients	Initial staging
Elstrom et al., 2008 [21]	Lymphoma: 196 (93%) - HL = 74 (35%) - NHL: 122 (43%) Myeloma or other suspected malignancies: 16 (7%)	U.S.	Conference abstract	73 patients	Staging (*n* = 61 scans) or therapy response assessment (*n* = 26 scans)
Bertaux, et al., 2020 [22]	CNS Lymphoma	France	Article	130 patients	Investigate suspicion of PCNS lymphoma
Mackintosh et al., 2019 [23]	Lymphoma: 196 (93%) - HL = 74 (35%) - NHL: 122 (43%) Myeloma or other suspected malignancies: 16 (7%)	Scotland	Conference abstract	212 patients	n/a

**Table 3:** Summary of study level prevalence of incidental finding.

Primary Diagnosis	Study	Prevalence of Incidental Findings	Prevalence of Clinically Significant Incidental Malignancy	Determination of Clinical Significance	Types of Incidental Malignancies	Secondary Outcomes
Myeloma or plasma cell disorders	[11]	Findings suspicious for cancer: n/a Total incidental findings: 163 findings in 94 patients (83.9%)	3 (1.8%) out of all incidental findings may be related to a clinically significant cancer	Biopsy not declared	1 small renal mass, 1 isodense nodule, 1 peritoneal carcinomatosis	n/a
	[12]	Findings suspicious for cancer: n/a Total incidental findings: 4 patients (13.8%)	3 patients (10.3%)	Biopsy not declared	1 prostate cancer, 1 parotid tumour, 1 lung cancer	Could not use SUVmax to determine the severity of disease. Positive correlation between painful lesions and SUV max.
	[13]	n/a	2 patients (2.4%)	Biopsy not declared	n/a	n/a
	[14]	Findings suspicious for cancer: n/a Total incidental findings: 9 patients (27.2%)	1 case of (3.0%)	Biopsy not declared	colorectal cancer	n/a
	[15]	Findings suspicious for cancer: n/a Total incidental findings: 73 patients (15.5%)	5 patients (1.1%)	Biopsy	1 lobular carcinoma of the breast, 1 prostate adenocarcinoma, 2 papillary thyroid carcinomas, 1 neuroendocrine tumour of the pancreas	Positive predictive value of 58.3% for incidental findings requiring further workup.
	[16]	Findings suspicious for cancer in myeloma patients: n/a Findings suspicious for cancer in MGUS patients: 0	11 patients (3.3%)	Biopsy not declared	3 thyroid gland carcinomas, 4 colon carcinomas, 1 breast carcinoma, 1 lung carcinoma, 1 lymphoma, 1 kidney carcinoma	All unexpectedly detected cancers were successfully treated due to early diagnosis.
	[17]	n/a	12 patients (1.6%)	Biopsy	7 tubular and/or tubulovillous adenomas, 2 lower gut plasmacytomas, 2 lower gut adenocarcinomas, 1 gastric plasmacytoma	No significant difference between SUV for abnormal and normal endoscopic findings.
Lymphoproliferativedisorders	[18]	Findings suspicious for cancer: n/a Total incidental thyroid findings: 52 patients (2.8%)	Malignant: 5 patients (0.3%) Benign: 27 patients (1.4%)	Histological confirmation	4 papillary thyroid cancer, 1 microinvasive follicular carcinoma	No statistically significant difference between the mean SUVmax of malignant (4.4) and benign (3.2) nodules. No significant difference between mean sizes of benign (23.7 mm) and malignant (23.6 mm) nodules.
	[19]	Incidental findings for suspicious abnormalities: 14 patients (4.8%)	Malignant: 8 patients (2.8%) Benign: 3 patients (1.0%)	Biopsy	4 colon cancers, 3 lung cancers, 1 pancreatic cancer	2 cases detected at staging, 6 cases detected after treatment. All 4 patients with colon carcinoma underwent curative surgery; successfully treated.
	[20]	Findings suspicious for cancer: n/a Total incidental thyroid findings: 223 findings in 70 patients (79.5%)	1 case (1.1%)	Biopsy not declared	papillary urothelial cell carcinoma	Patients without any findings showed better overall survival than patients with non-CTCL incidental findings, though not statistically significant.
	[21]	Findings suspicious for cancer: n/a Total incidental thyroid findings: 2 patients (2.3%)	1 case (1.4%)	Biopsy status unclear	Rectal cancer	n/a
	[22]	n/a	Malignant: 2 patients (1.5%) Benign: 5 patients (3.9%)	Biopsy status unclear	1 thyroid papillary carcinoma, 1 breast cancer	SUVMax of 9 was determined to differentiate systemic lymphoma from incidental findings (SUVmax < 9).
Myeloma or lymphoma	[23]	n/a	Malignant: 9 patients (4.2%) Benign: 4 patients (1.9%)	Pathological determination	2 adenocarcinomas, 4 precancerous polyps, 3 Thy3a	Surgical removal in patients with colon findings. No progression or relapse of the incidental findings at follow-up.

## Appendix A

The search strategy that was employed on Embase and MEDLINE is as follows:

1. exp hematologic neoplasms/or (leukemia* or lymphoma* or myeloma* or myelodysplastic* or myeloproliferative*).mp.
2. (leukemia* or lymphoma* or myeloma* or myelodysplastic* or myeloproliferative*).ab,ti.
3. exp Patients/
4. “patient* “.ab,ti.
5. 1 or 2
6. 3 or 4
7. 5 and 6
8. exp Positron Emission Tomography Computed Tomography/
9. (“PET-CT” or “positron emission tomography” or “computed tomography”).ab,ti.
10. 8 or 9
11. exp incidental finding/
12. “Incidental”.ab,ti.
13. 11 or 12
14. (“significan*” or “pathological* ajd3 finding*” or “clinical* relevant” or “clinical* adj3 finding*”).ab,ti.
15. 7 and 10 and 13 and 14
16. limit 15 to english language
